# Emodin Rescues Intrahepatic Cholestasis via Stimulating FXR/BSEP Pathway in Promoting the Canalicular Export of Accumulated Bile

**DOI:** 10.3389/fphar.2019.00522

**Published:** 2019-05-22

**Authors:** Xiao-Li Xiong, Yan Ding, Zhi-Lin Chen, Yao Wang, Pan Liu, Huan Qin, Li-Shan Zhou, Ling-Ling Zhang, Juan Huang, Lei Zhao

**Affiliations:** ^1^Department of Integrated Chinese and Western Medicine, Wuhan Children’s Hospital, Tongji Medical College, Huazhong University of Science and Technology, Wuhan, China; ^2^Department of Infectious Diseases and Immunology, Wuhan Children’s Hospital, Tongji Medical College, Huazhong University of Science and Technology, Wuhan, China; ^3^Department of Infectious Diseases, Union Hospital, Tongji Medical College, Huazhong University of Science and Technology, Wuhan, China; ^4^Department of Infectious Diseases, Renmin Hospital of Wuhan University, Wuhan, China; ^5^School of First Clinical Medicine, Hubei University of Chinese Medicine, Wuhan, China; ^6^Department of Clinical Laboratory, Wuhan Children’s Hospital, Tongji Medical College, Huazhong University of Science and Technology, Wuhan, China; ^7^Department of Pathology, Wuhan Children’s Hospital, Tongji Medical College, Huazhong University of Science and Technology, Wuhan, China

**Keywords:** emodin, cholestasis, bile salt export pump, signaling pathway, upregulation, downregulation

## Abstract

**Aim:**

Bile salt export pump (BSEP) have been confirmed to play an important role for bile acid canalicular export in the treatment of cholestasis. In this study, we investigated the stimulatory effect of emodin on BSEP signaling pathway in cholestasis.

**Methods:**

Cell and animal experiments were given different concentrations of emodin. The BSEP upstream molecule farnesoid X receptor was down-regulated by small interfering RNA (siRNA) technology or guggulsterones and up-regulated by lentivirus or GW4064. Real-time PCR and Western blotting was employed to detect the mRNA and protein levels of BSEP in LO2 cell, rat primary hepatocytes and liver tissue. Immunohistochemistry (IHC) was used to examine the expression of BSEP in liver tissues. Rat liver function and pathological changes of liver tissue were performed by biochemical test and hematoxylin and eosin (HE) staining.

**Results:**

Emodin could increase the mRNA and protein expression of BSEP and FXR. When down-regulating farnesoid X receptor expression with the siRNA or inhibitor guggulsterones, and up-regulating farnesoid X receptor expression with the lentivirus or agonist GW4064, emodin could increase the mRNA level of BSEP and FXR and the protein level of BSEP, FXR1, and FXR2. Emodin also had a notable effect on rat primary hepatocytes experiment, rat pathological manifestation, BSEP, FXR1, and FXR2 positive staining in liver tissues and the test of liver function.

**Conclusion:**

Emodin has a protective effect and a rescue activity on cholestasis via stimulating FXR/BSEP pathways in promoting the canalicular export of accumulated bile.

## Introduction

Intrahepatic cholestasis is characterized by disorders of bile formation, bile acid (BA) detoxification and its transport obstacles that are caused by the impairment of hepatocytes and cholangiocytes (Ding et al., 2008). As a common liver disease, cholestasis seriously affects the quality of life of patients, and if the disease cannot be effectively controlled, those patients will face liver fibrosis and biliary cirrhosis, and can ultimately die of liver failure (Fischler and Lamireau, 2014).

The cause of cholestasis is the bile secretion disorders of hepatocyte or bile duct epithelial cells and bile flow blockage by bile flow formation and excretion barriers, further leading to the retention of BA and other toxic substances, eventually causing liver cell damage and cholestatic liver disease (Wagner et al., 2009). The common incident parts involve the hepatocyte and intrahepatic bile duct system, and the mechanism is closely associated with the bile components, secretion, detoxification, and transportation (Hirschfield and Heathcote, 2009).

At present, the amount of cholestasis treatment drugs is limited, due to a lack of evidence for evidence-based medicine and clinical curative effect. Although ursodeoxycholic acid (UDCA) and *S*-adenosylmethionine are now supported by more and more evidence, and the clinical studies have confirmed that the two kinds of drugs in the treatment of cholestatic liver disease, such as primary biliary cirrhosis (PBC), primary sclerosing cholangitis and intrahepatic cholestasis of pregnancy, are effective, the drugs are not only expensive but also work slowly (Mato and Lu, 2007; Corpechot et al., 2008). Cholestasis as the main clinical manifestation of hepatitis syndrome is difficult to get rapid control of, and the efficacy is not cheap. On the other hand, although there has been evidence of certain clinical efficacy for glucocorticoid (such as dexamethasone, abbreviation for “DXM”) and other immunosuppressive agents, the obvious side effects of immunosuppression such as weight gain, hyperglycemia, osteoporosis, cataracts, and increased risk of opportunistic infections limit its clinical application (Purohit and Cappell, 2015). Therefore, it is urgent to develop new drugs with quick action and fewer side effects to treat cholestatic hepatitis.

Emodin (6-methyl-1,3,8-trihydroxyanthraquinone) is extracted from the traditional Chinese herb rhubarb as its main active ingredient. The molecular formula is C_15_H_10_O_5_ (Zhao et al., 2009). It has been confirmed that emodin has liver protection (Lee et al., 2012), anti-inflammation (Ding et al., 2008; Zhu et al., 2016), anti-virus (Ho et al., 2007), immune regulation (Kuo et al., 2001), promotion of gastrointestinal motility (Zhang et al., 2005), antioxidant (Li et al., 2013) and many other pharmacological effects. In the treatment of cholestatic hepatitis, emodin could alleviate the role of liver injury in cholestatic hepatitis caused by concanavalin A and alpha-naphthylisothiocyanate (ANIT) (Ding et al., 2008; Xue et al., 2015). More importantly, our previous work revealed that emodin could alleviate intrahepatic cholestasis by promoting the expression of liver farnesoid X receptor (FXR), small heterodimer partner (SHP), uridine diphosphate glucuronosyltransferase 2 family polypeptide B4 (UGT2B4), and bile salt export pump (BSEP), which are related to the synthesis, detoxification, and transportation process of Bas (Ding et al., 2016).

We have demonstrated that emodin played a protective role in intrahepatic cholestasis by promoting FXR signal pathways (Ding et al., 2016). However, the BSEP is the key regulator of BA canalicular export, and the molecular mechanism and target gene on alleviating intrahepatic cholestasis by emodin is still unknown. Therefore, the objective of this research was to confirm whether emodin could alleviate cholestasis via the BSEP signaling pathway. Therefore, this research used the LO2 cell line and ANIT-induced rat model to find how emodin interfered with the BSEP signaling pathway to alleviate intrahepatic cholestasis.

## Materials and Methods

### Reagents

Emodin (purity > 95%) was purchased from R&D (3811, Minneapolis, MN, United States). Cell Counting Kit-8 (CCK-8) was obtained from Dojindo (CK04, Mashikimachi, Japan). Dimethyl sulfoxide (DMSO) was purchased from Sigma-Aldrich China (D2650, Shanghai, China). ANIT were obtained from Alfa Aesar (STBD6070V, United States). Fetal bovine serum (FBS) was offered by Zhejiang Tianhang Biotechnology Co., Ltd. (141215, Hangzhou, China). RPMI-1640 medium were obtained from Gibco (8116524, Grand Island, NY, United States). DMEM/F-12 medium were purchased from HyClone (SH30023, Logan, United States). Phosphate-buffered saline (PBS), Trypsin 0.25% with EDTA and D-Hanks was purchased from Genom Biotechnology Co., Ltd. (GNM20012/GNM25200/GNM14170, Hangzhou, China). BCA Protein Assay Kit were bought from Beyotime (021317170407, Shanghai, China). Guggulsterone (purity ≥ 98%) and GW4064 (purity ≥ 95%) were obtained from Cayman Chemicals (71800-5/S2782, Ann Arbor, MI, United States). Rabbit anti-human FXR1 and FXR2 antibodies were, respectively, obtained from and Cell Signaling Technology (12295S/7098S, Danvers, MA, United States). Rabbit anti-human BSEP antibody and Cytokeratin 18 antibody was obtained from Abcam (ab140616/ab181597, Cambridge, MA, United States). Horseradish peroxidase (HRP)-labeled goat anti-rabbit IgG was obtained from Boster Immunoleader (BA1054, Wuhan, China). RNAiso Plus, PrimeScript^TM^ RT reagent Kit and SYBR Premix Ex Taq kit were purchased from TaKaRa (SD1410/RR036A/RR420A, Dalian, China). RIPA Lysis and Extraction Buffer, PMSF and electrochemiluminescence (ECL) kit were bought from Beyotime (AR0102-10/AR1182/EK1001, Shanghai, China). UDCA capsule was obtained from Dr. Falk Pharma GmbH (Freiburg, Germany). DXM was bought from Xinxiang Changle Pharmaceutical Company Ltd. (Xinxiang, China).

### Cell Culture

The normal human hepatocyte line LO2 was purchased from the Type Culture Collection of the Chinese Academy of Sciences, Shanghai, China. The human hepatocyte LO2 cell was maintained in an incubator at 37°C, 5% CO_2_ and saturated humidity in 1640 medium supplemented with 10% FBS (Guo et al., 2015a).

### Cytotoxic Effect of Emodin and Cell Morphology Observation

The cytotoxic effect of emodin was evaluated by the CCK-8 assay. Cell morphology was observed after treatment with emodin for 24 h and the procedure followed the previously published steps (Guo et al., 2015b; Wang et al., 2017).

### Cellular Model Establishment and Intervention

Groups were divided into a control group and UDCA (0.1 μg/ml), DXM (0.785 ng/μl) and emodin (0.02 μg/ml, 0.04 μg/ml and 0.08 μg/ml) groups. With cells passage in 6-, 12-, or 96-well plates for 24 h and culturing to 70% density, the supernatants were removed and guggulsterone (1 μM) or GW4064 (1 μM) was added to the wells, excluding the control group. The cell-culture medium was replaced 24 h later, and the guggulsterone/GW4064 was diluted in 1640 medium. After 24 h, the cells were harvested for quantitative real-time PCR and Western blotting.

### Small Interfering (si) RNA Transfection in LO2 Cells

The FXR siRNA (sense 5′-CAAGTGACCTCGACAACAA-3′) for human was synthesized by RiboBio Co., Ltd. (Guangzhou, China). LO2 cells were seeded onto 6-well plates and transfected with siRNA duplexes using Lipofectamine 2000 (Invivogen, San Diego, CA, United States) according to manufacturer’s instructions. The medium was replaced 6 h later, and then the cells were grown for an additional time up to 48 h. At the time point of 24 h before harvest, the siRNA-intervened cells were treated with emodin, UDCA or DXM.

### Lentivirus Transfection in LO2 Cells

The FXR and negative normal lentiviral vectors were constructed by GeneChem Co., Ltd., Shanghai, China. GV367-FXR/NC-enhanced green fluorescent protein (EGFP) was transfected into LO2 cell lines, and viral supernatant was harvested after 48 h (2 × 10^8^ transducing units [TU]/ml). LO2 cells were seeded onto 96- or 6-well plates and transfected with lentivirus with a multiplicity of infection of 50 according to manufacturer’s instructions. The medium was replaced 6 h later, and then the cells grew for an additional time up to 72 h. Then, the lentivirus-intervened cells were treated with emodin, UDCA or DXM for another 24 h.

### Animal Model Establishment and Specimen Collection

Specific pathogen-free neonatal Sprague-Dawley rats (60–80 g) were obtained from Hubei Provincial Centers for Disease Control, including 21 female rats and 21 male rats. All rats were kept under constant housing conditions with 22°C, 60% relative humidity, and a 12-h light/dark cycle and had free access to water and food throughout the experiment (Jin et al., 2013; Du et al., 2016). The animal experiment number was SCXK (HUBEI) 2015-0018. The study was reviewed and approved by the Research Ethics Committee of Tongji Medical College, Huazhong University of Science and Technology (Li et al., 2016; Yang et al., 2016). After feeding for 3 days for adaptation, 42 rats were equally divided into seven groups, i.e., emodin (80 mg/kg), emodin (40 mg/kg), emodin (20 mg/kg), UDCA, DXM, model and control groups. Each group included six rats with 3 males and 3 females. Emodin was prepared to 0.4%, 0.2%, and 0.1% suspensions by sodium carboxymethylcellulose. UDCA was prepared to 0.3% suspension by water. Dexamethasone was dissolved in water with the concentration of 0.009%. ANIT was dissolved in sesame oil with the concentration of 0.25%. The intervention cycle for the model was 7 days for emodin (80 mg/kg, 40 mg/kg, 20 mg/kg), UDCA (60 mg/kg) and dexamethasone (1.8 mg/kg) groups, and 0.25% ANIT (50 mg/kg) was given to the six groups on the fifth day through administration by gavage. Animals were terminated by cervical dislocation after 48 h of ANIT treatment. During this time, the rats were fasted for the last 12 h and anesthetized for the removal of 2 ml of eyeball blood before their death, and all the rats were anesthetized through intraperitoneal injection with 10% chloral hydrate (0.3 ml/100 g). The serum was stored at -20°C. The right liver was immediately snap frozen in liquid nitrogen and stored at -80°C, while the left lobe tissue was fixated in 4% formaldehyde and embedded in paraffin.

### Extraction, Cultivation, and Treatment of Rat Primary Hepatocytes

Adult male SD rats (6–8 weeks) weighing 180–200 g were chose to extract hepatocytes, and we use two-step collagenase digestion method and cultivated according to published steps (Klaunig et al., 1981). Extract hepatocytes was maintained in an incubator at 37°C, 5% CO_2_ and s cultured humidity in 1640 medium supplemented with 20% FBS, We used immunofluorescence to detect CK-18 protein in cells to identify whether the extracted cells were rat primary hepatocytes (Banaudha et al., 2010). Then, the hepatocytes were planted in 6-well plates. When the cell cultivated reaching 70% density after 12 h, they were treated with emodin (0.02 μg/ml, 0.04 μg/ml, and 0.08 μg/ml), UDCA (0.1 μg/ml), DXM (0.785 ng/μl). After 24 h, each group of cells was harvested for quantitative real-time PCR and Western blotting, respectively.

### Quantitative Real-Time PCR for mRNA Expression Measurement

Following our previous steps (Zhou et al., 2013), total RNA from liver tissues and cells were isolated using RNAiso Plus following the manufacturer’s protocol. The cDNAs were produced with PrimeScript RT reagent kit and incubated at 37°C for 15 min and 85°C for 5 s. Real-time PCR reactions were done using a StepOne Plus device (Applied Biosystems) at 95°C for 10 s followed by 40 cycles of 95°C for 5 s and 60°C for 20 s according to instruction of the SYBR Premix Ex Taq kit. Data were analyzed by 2^-ΔΔCt^ method. All primers were synthesized by TSINGKE (Wuhan, China). The sequences of all primers are listed in Table 1.

**Table 1:** Sequences of primers for reverse transcription and real-time polymerase chain reaction amplification.

Gene		Primer sequence (5′ → 3′)	bp
FXR	Forward primer	AAGTGACCTCCACGACCAAGC	21
(human)	Reverse primer	TCCGCTGAACGAAGGAACAT	20
FXR	Forward primer	AAGAGATGGGAATGTTGGCTG	21
(rat)	Reverse primer	CTCCCTGCATGACTTTGTTGTC	21
BSEP	Forward primer	ATGTTGACGGGATTCGCTTC	20
(human)	Reverse primer	CCACTCCAATCCCAGCAACT	20
BSEP	Forward primer	CAACTGCTGGACCGAC AACC	20
(Rat)	Reverse primer	CATCCACTGCTCCCAACAA AC	20
GAPDH	Forward primer	AGGTCCACCACTGACACGTT	20
(human)	Reverse primer	GCCTCAAGATCATCAGCAAT	20
GAPDH	Forward primer	ACAGCAACAGGGTGGTGGAC	20
(rat)	Reverse primer	TTTGAGGGTGCAGCGAACTT	20


### Western Blotting for Protein Expression Measurement

Abiding by our previous steps (Li et al., 2017), nucleus protein and total protein were extracted from the liver tissues and hepatocytes, respectively. The protein concentration was determined using BCA method. To each tube, an equivalent volume of 2× sodium dodecyl sulfate (SDS) loading buffer (100 mM Tris-HCl, pH 6.8, 4% SDS, 20% glycerine, 10% 2-mercaptoethanol, and 0.2% bromophenol blue) was added and mixed again. The mixtures were then denatured at 95°C for 10 min, and approximately 30 mg of the protein mixture was loaded and separated in each well on 10% SDS-polyacrylamide electrophoresis gels. After separation for approximately 80 min, the proteins were transferred to polyvinylidene difluoride (PVDF) membranes, and the membranes were saturated and blocked with 5% fat-free milk at 37°C for 1 h. Membranes were probed with rabbit polyclonal anti-rat-FXR1 (1:1000), FXR2 (1:500), BSEP (1:500) and then with horseradish peroxidase-conjugated secondary immunoglobulin IgG (1:1000). The membranes were then treated with an enhanced chemiluminescence reagent (Amersham, Piscataway, NJ, United States), and the signals were detected by exposure of the membranes to X-ray films (Kodak, Rochester, NY, United States). The relative signal intensity was quantified by densitometry with Gel pro3.0 image software (Media Cybernetics, Silver Spring, MD, United States) on an IBM-compatible personal computer.

### Immunohistochemistry (IHC) for Detecting BSEP Expression in Liver Tissue

The procedure followed our previous steps (Jin et al., 2015). The liver tissue specimens were cut into 10 μm sections after dewaxing and hydrating. The sections were incubated in 3% H_2_O_2_/methanol to eliminate endogenous peroxidase activity. Then, the sections were incubated with normal goat serum for 10 min and incubated with FXR1 (1:200), FXR2 antibody (1:75) and BSEP (1:40) overnight at 4°C and biotin-conjugated goat anti rabbit IgG (1:200) at 37°C for 45 min. They were rinsed again with PBS and incubated with horseradish peroxidase-labeled streptavidin at 37°C. The samples were developed with diaminobenzidene (DAB) and stained with hematoxylin. After being rinsed with distilled water and dehydrated, the sections were made transparent and mounted for microscope examination. After the immunohistochemical analysis, IPP software (image-pro plus 6.0) was used to analyze the optical density of the images as described previously.

### Biochemical Tests

The serum total bilirubin (TBIL), direct bilirubin (DBIL), alanine aminotransferase (ALT), aspartate aminotransferase (AST), alkaline phosphatase (ALP), γ-glutamyl transpeptidase (GGT), and total bile acids (TBA) were assayed by Aeroset Fully-auto Chemistry Analyzer provided by Abbott Co LTD.

### Histomorphology

After fixation in 4% formaldehyde, tissues were embedded in paraffin and cut in serial sections of 4 μm for hematoxylin and eosin (HE) staining as our previous studies (Huang et al., 2013).

### Statistical Analysis

The statistical analyses were conducted with SPSS 12.0 software. Data were expressed as the mean ± SEM. The comparisons of the measurement data between the groups were performed with one-way ANOVA tests and Student’s *t*-tests. Statistical significance was defined at *p* < 0.05 (Ding et al., 2015).

## Results

### Effects of Emodin on the BSEP Pathway in LO2 Cell

**FIGURE 1:**
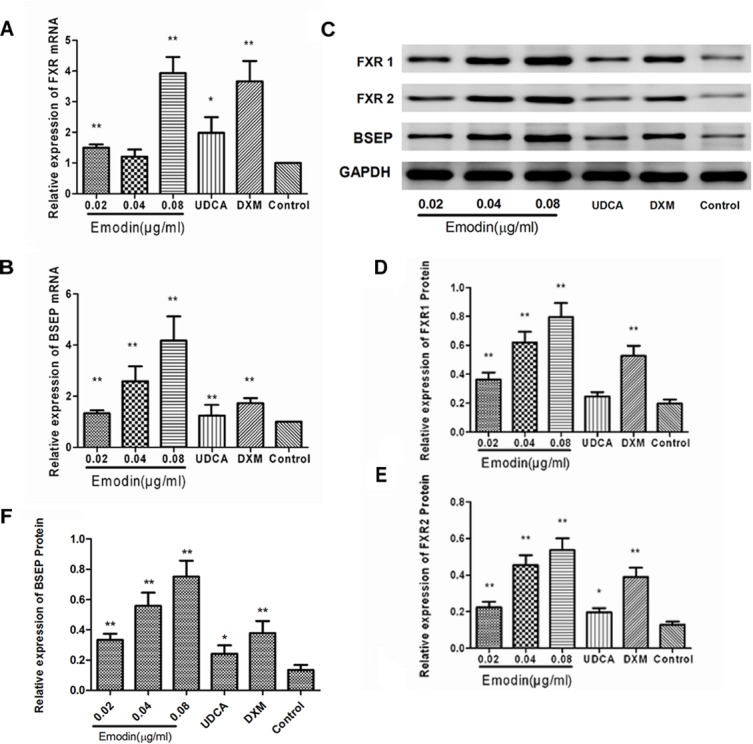
Effect of emodin on the expression of FXR and BSEP in normal LO2 cells. The mRNA levels of FXR **(A)** and BSEP **(B)** were detected by RT-PCR. The protein levels of FXR1, FXR2, and BSEP were measured by Western blot **(C–F)**. Values are the means ± SD (*n* = 3, ^∗^*P* < 0.05 compared to the control group; ^∗∗^*P* < 0.01 compared to the control group, as determined by Student’s *t*-test).

Based on the CCK8 assay, pretreatment of unstimulated LO2 cells with prepared solution of emodin at 0.02 μg/ml, 0.04 μg/ml, and 0.08 μg/ml for 24 h did not significantly affect cell viability. Therefore, we chose emodin at 0.02 μg/ml, 0.04 μg/ml, and 0.08 μg/ml to treat cells for 24 h. Compared with the control group, the mRNA expressions of BSEP and FXR were significantly elevated in the emodin group (*p* < 0.05 or 0.01) (Figure 1A,B). The protein expressions of BSEP, FXR1 and FXR2 were also significantly increased in the emodin groups (*p* < 0.05 or 0.01); UDCA could increase the mRNA and protein level of BSEP (*P* < 0.01 or *P* < 0.05) (Figure 1C–F). Compared with the control group, emodin could notably increase the mRNA level of FXR, as well as the protein level of FXR1 and FXR 2 (*P* < 0.01).

### Effects of Emodin on the BSEP Pathway in LO2 Cell After Guggulsterone Stimulation

**FIGURE 2:**
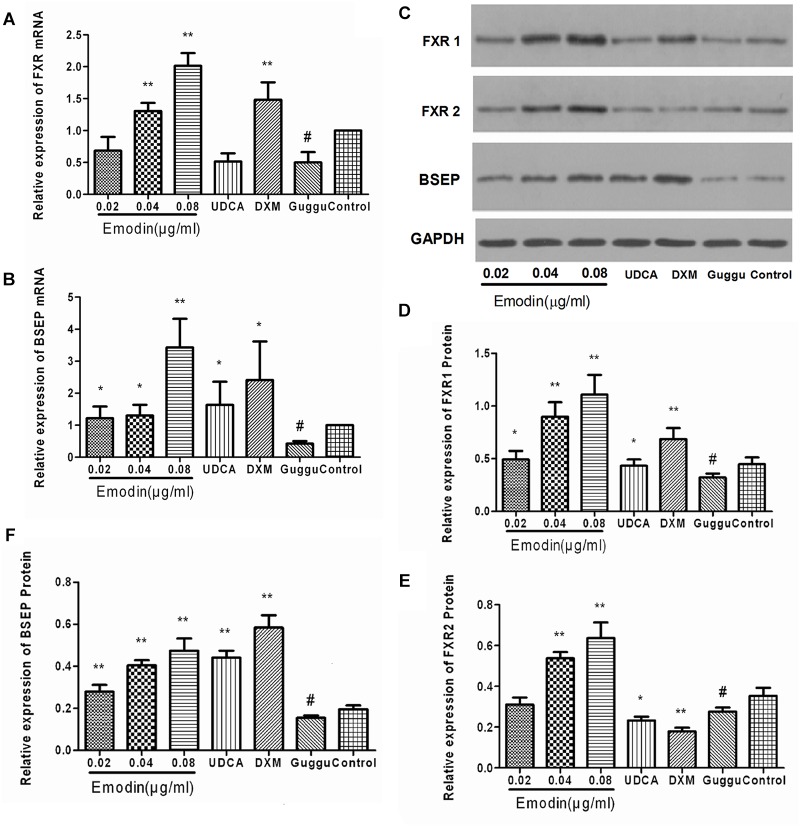
Effect of emodin on the expression of FXR and BSEP in LO2 cell after guggulsterone stimulation. The mRNA levels of FXR **(A)** and BSEP **(B)** were detected by RT-PCR. The protein levels of FXR1, FXR2, and BSEP were measured by Western blot **(C–F)**. Values are the means ± SD (*n* = 3, ^∗^*P* < 0.05 compared to the guggulsterone group; ^∗∗^*P* < 0.01 compared to the guggulsterone group; ^#^*P* < 0.01 compared to the control group, as determined by Student’s *t*-test).

The LO2 cells were interfered with using guggulsterones. Compared with the control group, the mRNA expressions of BSEP and FXR were significantly decreased in the guggulsterones group (*p* < 0.01), while compared with the guggulsterones group, the mRNA expressions of BSEP and FXR were significantly elevated in the emodin groups (*p* < 0.05 or 0.01) (Figure 2A,B). The protein expressions of BSEP, FXR1, and FXR2, were also significantly lowered by guggulsterones (*p* < 0.01), and compared with the guggulsterones group, the protein expressions of BSEP, UGT2B4, FXR1, and FXR2 were significantly elevated in the emodin groups (*p* < 0.05 or 0.01) (Figure 2C–F).

### Effects of Emodin on the BSEP Pathway in LO2 Cell After GW4064 Stimulation

**FIGURE 3:**
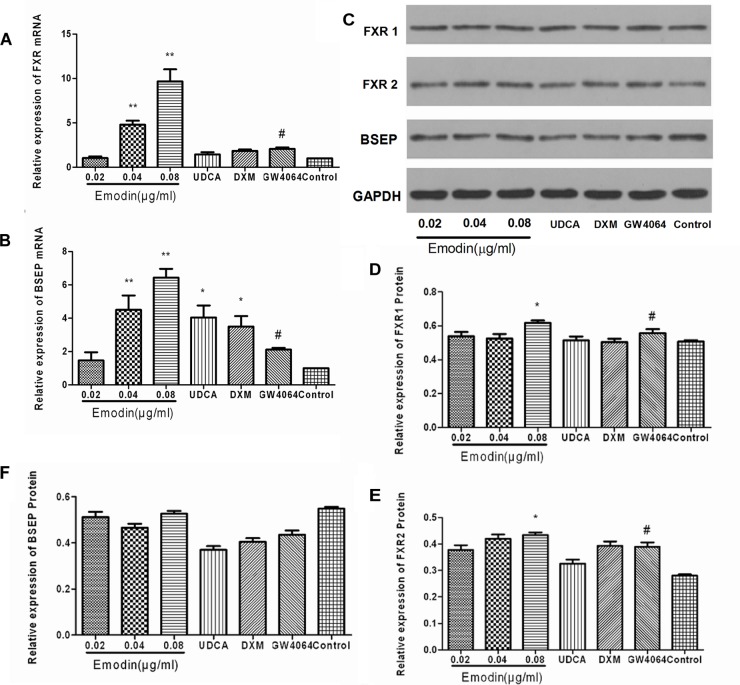
Effect of emodin on the expression of FXR and BSEP in LO2 cell after GW4064 stimulation. The mRNA levels of FXR **(A)** and BSEP **(B)** were detected by RT-PCR. The protein levels of FXR1, FXR2, and BSEP were measured by Western blot **(C–F)**. Values are the means ± SD (*n* = 3, ^∗^*P* < 0.05 compared to the GW4064 group; ^∗∗^*P* < 0.01 compared to the GW4064 group; ^#^*P* < 0.01 compared to the control group, as determined by Student’s *t*-test).

The LO2 cells were interfered with using GW4064. Compared with the control group, the mRNA expressions of BSEP and FXR were significantly increased in the GW4064 group (*p* < 0.01), while compared with the GW4064 group, the mRNA expressions of BSEP and FXR were significantly elevated in the emodin groups (*p* < 0.05 or 0.01) (Figure 3A,B). The protein expressions of FXR1 and FXR2 were also significantly elevated in the GW4064 group (*p* < 0.01), and compared with the GW4064 group, the protein expressions of FXR1 and FXR2 were significantly elevated in the emodin group (*p* < 0.05) (Figure 3C–F).

### Effect of Emodin on the BSEP Pathway in LO2 Cells When FXR Was Knocked Down by siRNA

**FIGURE 4:**
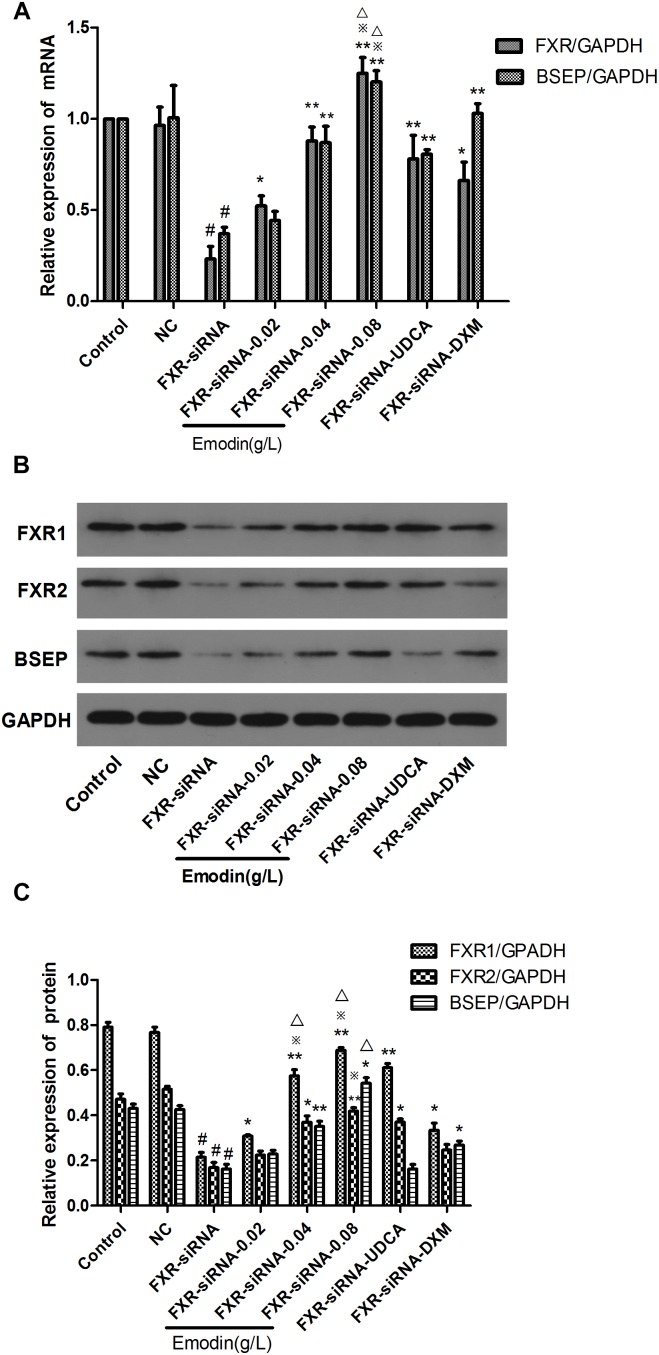
Effect of emodin on the expression of FXR and BSEP after FXR was knocked down by siRNA. The mRNA levels of FXR and BSEP were detected by RT-PCR **(A,B)**. The protein levels of FXR1, FXR2, and BSEP were measured by Western blot **(C)**. Values are the means ± SD (*n* = 3, ^#^*P* < 0.01 compared to the control group, ^∗∗^*P* < 0.01 compared to the FXR-siRNA group; *P* < 0.05 compared to the FXR-siRNA group; ^∗^*p* < 0.05 compared to the UDCA group; ^
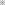
^ *p* < 0.05 compared to the DXM group, as determined by Student’s *t*-test).

We used siRNA to down-regulate the expression of FXR in LO2 cells for 24 h. Then, the cells were treated with emodin (0.02, 0.04 and 0.08 μg/ml), UDCA or DXM for 24 h. Compared with the control group, the mRNA expressions of BSEP and FXR were significantly decreased in the FXR-siRNA group (*p* < 0.01) (Figure 4A), and the protein expressions of BSEP, FXR1, and FXR2 were also significantly lowered in the FXR-siRNA group (*p* < 0.01) (Figure 4B,C). While compared with the FXR-siRNA group, the mRNA expressions of BSEP, FXR1, and FXR2 were significantly elevated in the emodin groups (*p* < 0.05 or 0.01) (Figure 4A). While compared with the FXR-siRNA group, the protein expressions of BSEP, FXR1, and FXR2 were also significantly elevated in the emodin groups (*p* < 0.05 or 0.01) (Figure 4B,C).

### Effect of Emodin on the BSEP Pathway in LO2 Cells After FXR Over-Expression by Lentivirus Transfection

**FIGURE 5:**
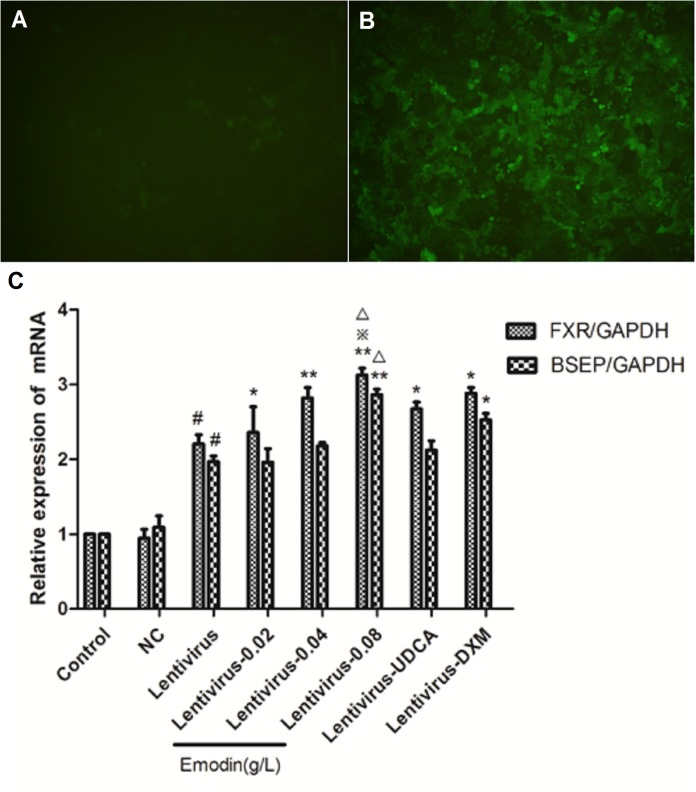
The expression of GFP was observed with a fluorescence microscope after lentivirus was introduced into LO2 cells for 48 h **(A)** and 72 h **(B)**. The mRNA levels of FXR and BSEP were detected by RT-PCR **(C)**. (*n* = 3, ^#^*P* < 0.01 compared to the control group, ^∗∗^*P* < 0.01 compared to the lentivirus group; ^∗^*P* < 0.05 compared to the lentivirus group; ^
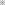
^*p* < 0.05 compared to the UDCA group, as determined by Student’s *t*-test).

**FIGURE 6:**
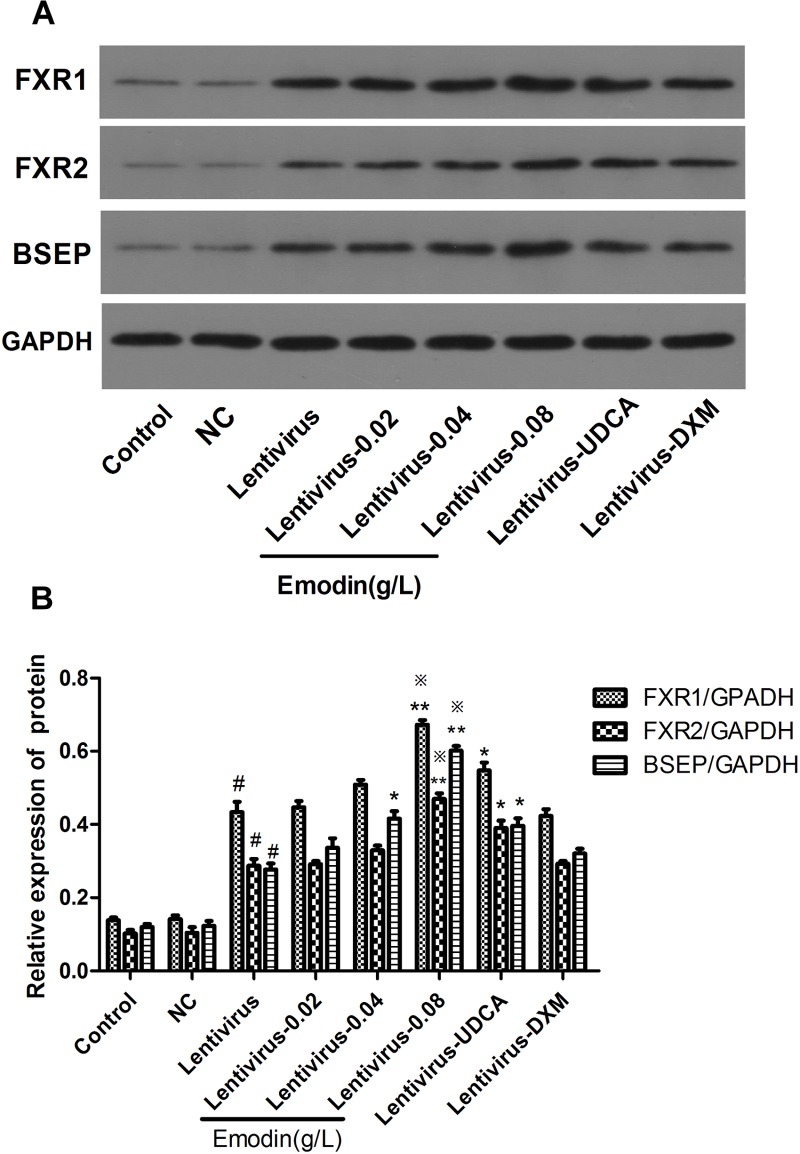
The protein levels of FXR1, FXR2, and BSEP were detected by Western blotting **(A,B)**. Values are the means ± SD (*n* = 3, ^#^*P* < 0.01 compared to the control group, ^∗∗^*P* < 0.01 compared to the lentivirus group; ^∗^*P* < 0.05 compared to the lentivirus group; ^
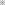
^*p* < 0.05 compared to the UDCA group, as determined by Student’s *t*-test).

We made the FXR lentiviral vector GV273 and transfected LO2 cells. GFP (green fluorescent protein) was observed with a fluorescence microscope after 48 h and 72 h (Figure 5A,B). Then, the cells were treated with emodin (0.02, 0.04 and 0.08 μg/ml), UDCA or DXM for 24 h. Compared with the control group, the mRNA expressions of BSEP and FXR were significantly increased in the lentivirus-up group (*p* < 0.01) (Figure 5C), and the protein expressions of BSEP, FXR1, and FXR2 were also significantly elevated in the lentivirus-up group (*p* < 0.01) (Figure 6A,B). Compared with the lentivirus-up group, the mRNA expressions of BSEP and FXR were significantly elevated in the emodin groups (*p* < 0.05 or 0.01) (Figure 5C), while the protein expressions of BSEP, FXR1, and FXR2 were also significantly elevated in the emodin groups (*p* < 0.05 or 0.01) (Figure 6A,B).

### Effect of Emodin on Serum Biochemical Indicators

In this experiment, we set three double ratio concentration 20 mg/kg, 40 mg/kg, and 80 mg/kg. The acute toxicity study for anthraquinones (emodin) showed LD_50_ values of 8.6 g/kg and 3.8 g/kg in male and female mice, respectively (Djimeli et al., 2017), which is much higher than the three concentrations in our experiment.

Indeed, as is showed in Table 2, there is no dose–response in emodin. For ALT, AST, DBIL, TBIL, GTT, and ALP, the superior rank was: 40 mg/kg > 20 mg/kg > 80 mg/kg. For TBA, the superior rank was: 40 mg/kg > 80 mg/kg > 20 mg/kg. So, the optimum concentration for emodin may be 40 mg/kg and the underlying causes deserve further studies.

**Table 2:** Effect of emodin on liver function tests.

Group	ALT (U/L)	AST (U/L)	DBIL (μmol/L)	TBIL (μmol/L)	GTT (U/L)	ALP (King unit/100 ml)	TBA (μmol/L)
Emodin (20 mg/kg)	55.4 2.7^*@%^	67.6 3.4^*@%^	41.9 2.7^*#^	73.1 4.6^*@%^	47.3 2.3^*@&^	46.3 2.3^*@%^	142.9 7.1^*@%^
Emodin (40 mg/kg)	48.6 3.2^*@% 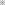 ^	62.0 3.5^*@% 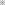 ^	39.4 3.1^*#^	59.1 4.6^*@%^	42.4 3.0^*@%^	42.9 2.4^*@%^	99.5 3.5^*@% 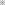 ^
Emodin (80 mg/kg)	60.0 3.8^*@&^	74.4 3.0^*@&^	44.3 2.4^*^	78.0 4.4^*@&^	51.7 3.1^*@^	50.1 2.0^*@&^	120.4 4.2^*@% 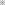 ^
UDCA	69.1 3.3^*@^	82.8 3.7^*#^	45.3 3.5^*^	85.7 4.3^*@^	54.7 3.0^*#^	54.5 3.3^*#^	157.1 5.0^*@%^
DXM	72.1 3.1^*@^	86.3 3.5^*^	48.5 3.1^*^	95.3 5.0^*#^	58.1 2.6^*^	58.9 1.9^*#^	186.6 5.9^*@^
Model	85.1 1.8^*^	95.8 3.7^*^	52.3 3.1^*^	114.6 5.6^*^	66.2 2.8^*^	66.9 2.6^*^	221.3 8.7^*^
Control	24.8 3.3	27.5 2.4	19.8 1.9	44.1 3.4	15.7 1.4	30.2 1.1	47.4 2.2


*Data were shown as mean ± SD. *n* = 6. (^∗^*P* < 0.01 vs. control group; ^#^*P* < 0.05, ^@^*P* < 0.01 vs. model group; ^&^*P* < 0.05, %*P* < 0.01 vs. DXM group; ^
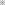
^*P* < 0.01 vs. UDCA group.)*

As shown in Table 2, when compared with the model group, emodin had a significant effect on decreasing ALT, AST, TBIL, DBIL, ALP, GGT, and TBA levels (*P* < 0.05 or *P* < 0.01). On ALT, emodin (20 mg/kg) and emodin (80 mg/kg) had a similar effect with UDCA. Emodin (40 mg/kg) was most effective on AST, ALP, and DBIL (*P* < 0.01), which was inferior to UDCA. Emodin (80 mg/kg and 40 mg/kg) had a notable effect on TBIL (*P* < 0.01), and UDCA showed a similar effect on TBIL level as the emodin (20 mg/kg) group. It was significant that emodin (20 mg/kg, 40 mg/kg, 80 mg/kg) had an effect on GGT (*P* < 0.05 or 0.01), and emodin (40 mg/kg) was markedly more effective (*P* < 0.01) than the UDCA group on GGT. There is no dose–response in emodin. For ALT, AST, DBIL, TBIL, GTT, and ALP, the superior rank was: 40 mg/kg > 20 mg/kg > 80 mg/kg. And for TBA, the superior rank was: 40 mg/kg > 80 mg/kg > 20 mg/kg. So, the optimum concentration for emodin may be 40 mg/kg.

### Effects of Emodin on BSEP Pathway in aRat Model With Cholestatic Hepatitis

**FIGURE 7:**
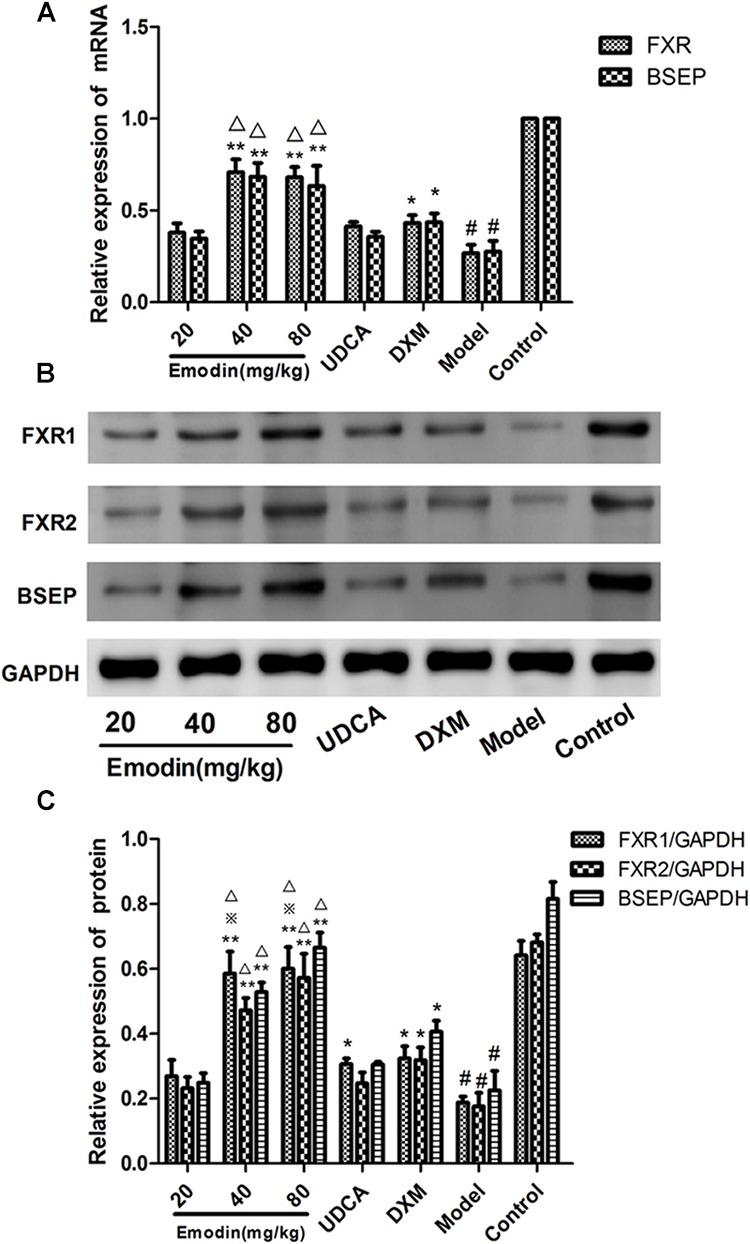
Effect of emodin on the SHP-BSEP signaling pathway in ANIT-treated rats. The mRNA levels of FXR and BSEP were measured by real-time PCR **(A)**. The protein levels of FXR1, FXR2, and BSEP were detected by Western blotting **(B,C)**. Data were expressed as the mean ± SD. *n* = 3. (^#^*P* < 0.01 compared to the control group, ^∗∗^*P* < 0.01 compared to the model group; ^
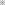
^ *p* < 0.05 compared to the UDCA group; ^Δ^*p* < 0.05 compared to the DXM group, as determined by Student’s *t*-test).

Compared with the control group, the mRNA levels of BSEP and FXR in the model group were decreased significantly (*P* < 0.01). The mRNA expression of BSEP and FXR was affected by emodin treatments when compared with model group (*P* < 0.01 or 0.05) (Figure 7A). Compared with the control group, the protein expressions of BSEP, FXR1, and FXR2 were significantly decreased in the model group (*p* < 0.01). While compared with the model group, the protein expressions of BSEP, FXR1, and FXR2 were significantly elevated in the emodin groups (*p* < 0.01) (Figure 7B,C). Although UDCA showed a positive effect on the mRNA and protein expression of BSEP, FXR1, and FXR2 when compared with model group (*p* < 0.01 or 0.05), emodin (40 mg/kg) and emodin (80 mg/kg) groups presented better improvement than that in UDCA and DXM group (*P* < 0.05 or 0.01).

### Effects of Emodin on Molecules of BSEP Pathway in Rat Primary Hepatocytes

**FIGURE 8:**
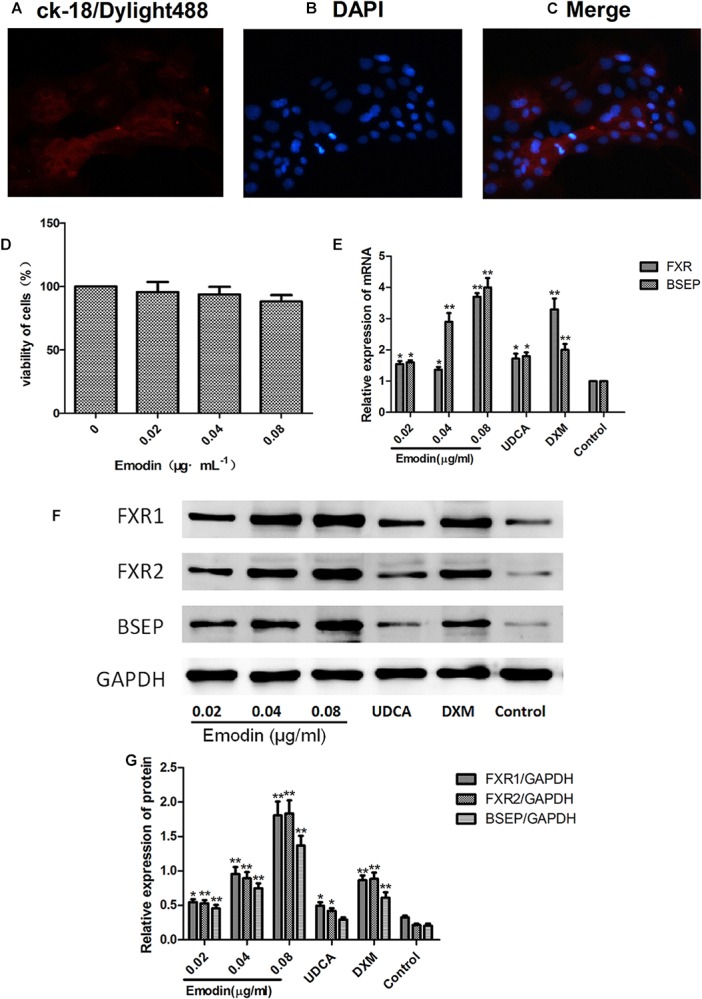
Effects of emodin on molecules of BSEP pathway in rat primary hepatocytes. Immunofluorescence was used to detect CK-18 protein in hepatocytes, and the red fluorescence molecule Dylight488 in primary hepatocytes was observed under a fluorescence microscope **(A–C)**. Cytotoxicity of emodin on primary hepatocytes was determined by CCK8 assay **(D)**. The mRNA levels of FXR and BSEP were detected by qRT-PCR **(E)**. The protein levels of FXR1, FXR2, and BSEP were detected by Western blotting **(F,G)**. Data are shown as the mean ± SD. *n* = 5. (^∗^*P* < 0.05 compared to the control group; ^∗∗^*P* < 0.01 compared to the control group).

Rat primary hepatocytes were used to study the effects of emodin on cholestasis. To demonstrate whether the extracted cells were rat primary hepatocytes, we used immunofluorescence to detect CK-18 protein in hepatocytes and observed the red fluorescent molecule Dylight488 in primary hepatocytes by fluorescence microscope (Figure 8A–C). As shown in Figure 8D, compared with the untreated cells, the emodin group showed significantly elevated mRNA levels of FXR and BSEP. More importantly, the increases in the mRNA levels of FXR in the 0.08 μg ⋅ ml^-1^ emodin group were more significant than those in the UDCA groups (Figure 8D,E). The protein expression of FXR and BSEP were also significantly increased, and there were no obvious increases in protein expression in the UDCA groups (Figure 8F,G). Consistent with the results of the LO2 hepatocyte cell line, the experimental results of primary hepatocytes showed FXR/BSEP pathways were markedly up-regulated in the 0.04 and 0.08 μg ⋅ ml^-1^ emodin groups *in vitro*.

### Effects of Emodin on Liver Morphology by HE Staining

**FIGURE 9:**
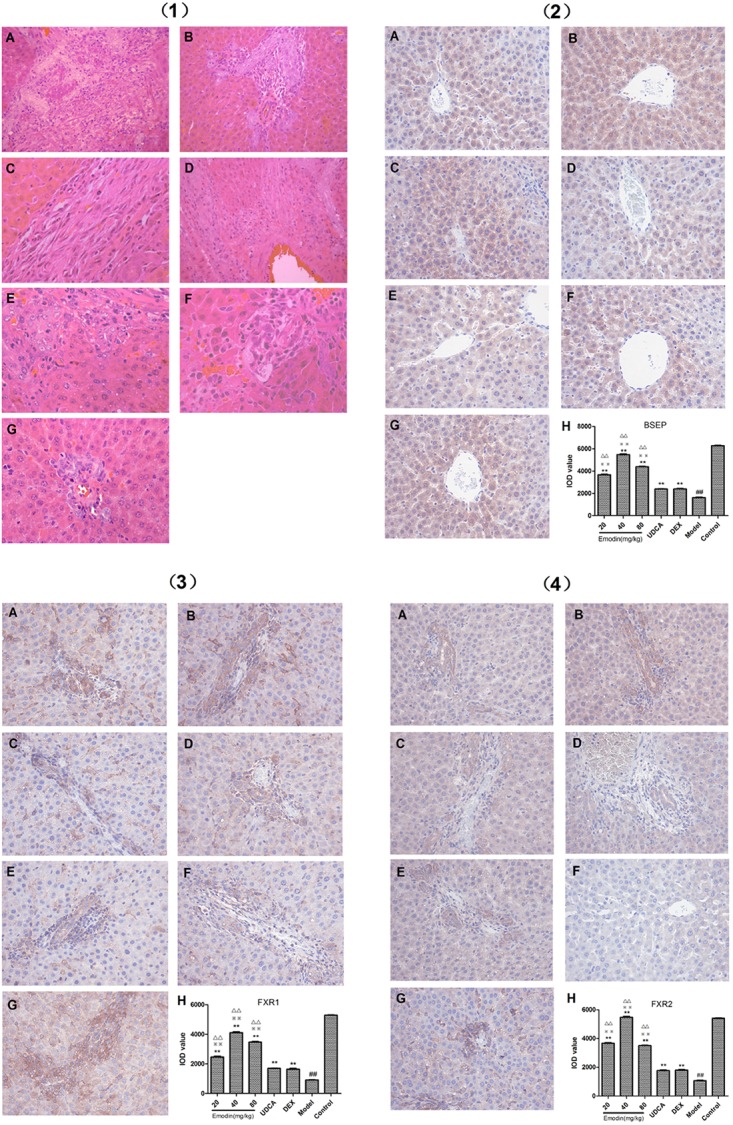
**(1)** Effect of emodin on pathological manifestation of hepatic tissue by HE staining at 400× magnification (**A**: emodin 20 mg/kg group; **B**: emodin 40 mg/kg group; **C**: emodin 80 mg/kg group; **D**: UDCA group; **E**: DXM group; **F**: model group; **G**: control group). **(2)** Effect of emodin on BSEP expression was examined with IHC at 400× magnification (**A**: emodin 20 mg/kg group; **B**: emodin 40 mg/kg group; **C**: emodin 80 mg/kg group; **D**: UDCA group; **E**: DEX group; **F**: model group; **G**: control group. Values are the means ± SD. *n* = 3, ^##^*P* < 0.01 compared to the control group, ^∗∗^*P* < 0.01 compared to the model group; ^
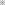

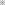
^*p* < 0.01 compared to the UDCA group; ^ΔΔ^*p* < 0.01 compared to the DXM group, as determined by Student’s *t*-test). **(3)** Effect of emodin on FXR1 expression was examined with IHC at 400× magnification (**A**: emodin 20 mg/kg group; **B**: emodin 40 mg/kg group; **C**: emodin 80 mg/kg group; **D**: UDCA group; **E**: DXM group; **F**: model group; **G**: control group. Values are the means ± SD. *n* = 3, ^##^*P* < 0.01 compared to the control group, ^∗∗^*P* < 0.01 compared to the model group; ^
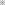

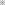
^*p* < 0.01 compared to the UDCA group; ^ΔΔ^*p* < 0.01 compared to the DXM group, as determined by Student’s *t*-test). **(4)** Effect of emodin on FXR2 expression was examined with IHC at 400× magnification (**A**: emodin 20 mg/kg group; **B**: emodin 40 mg/kg group; **C**: emodin 80 mg/kg group; **D**: UDCA group; **E**: DEX group; **F**: model group; **G**: control group. Values are the means ± SD. *n* = 3, ^##^*P* < 0.01 compared to the control group, ^∗∗^*P* < 0.01 compared to the model group; ^
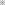

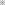
^*p* < 0.01 compared to the UDCA group; ^ΔΔ^*p* < 0.01 compared to the DXM group, as determined by Student’s *t*-test).

As shown in Figure 9(1), the hepatic tissue in the control group showed regular arrangement of hepatic lobules and cells and intact epithelial cells of bile duct. The pathological changes in the model group were typically showing significant swelling of hepatic cells, swelling of cytoplasm, uniformed nucleus and a strongly stained nucleolus. Moreover, in the model group, many punctiform or focused necrotic zones were shown in the hepatic tissue and Kupffer’s cell could also be observed. At the same time, the bile duct showed a narrower canal and bile thrombus and necrotic cells. The pathological impairment in the DXM group appeared more serious in hepatic tissue. However, in emodin groups, the pathological changes were less than the model group and the manifestations in the UDCA group were similar to those in emodin (20 mg/kg) group.

### Effect of Emodin on BSEP Protein Expression Examined by IHC in Liver Tissues

As shown in Figure 9(2)–(4) the rate of BSEP, FXR1, and FXR2 positive staining in the model group significantly decreased (*P* < 0.01) compared with the control group. With emodin treatment, the rates of BSEP-, FXR1-, and FXR2-positive staining could be increased notably (*P* < 0.01). The BSEP-, FXR1-, and FXR2-positive staining rates in UDCA and DXM groups were higher than that in the model group (*P* < 0.01). Compared with UDCA and DXM group, the BSEP-, FXR1-, and FXR2-positive rates in the emodin group were markedly higher (*P* < 0.01).

## Discussion

As a member of adenosine triphosphate (ATP)-binding cassette (ABC) transporters, BSEP is encoded by the gene ABCB11 (Meier et al., 2006). It is mainly expressed on the hepatocytes canalicular membranes and utilizes energy that from ATP hydrolysis to transport intracellular substrates into the extracellular compartment. So BSEP is primarily responsible for the BA excretion (Cheng et al., 2016) and translocates bile salt from hepatocytes into the canaliculus and the multidrug resistance protein 3 (MDR3) translocates phosphatidylcholine from the cytoplasmic to the outer leaflet of the canalicular membrane. Bile salt in the canaliculus facilitates the release of phosphatidylcholine from the outer leaflet and forms phosphatidylcholine-bile salt-mixed micelles, which is critical for the solubilization of biliary cholesterol. Moreover, phosphatidylcholine-bile salt-mixed micelles reduce the toxic activity of bile salts against the bile ducts. Therefore, the proper and coordinated functioning of BSEP and MDR3 is critical for mixed micelle formation and can lead to a decreased solubility of biliary cholesterol and consequently to cholestasis (Mahdi et al., 2016).

Recent studies have shown that the nuclear receptor FXR is the central molecule in the metabolism of BAs and is currently considered as key target in the treatment of cholestasis (Stahl et al., 2008; Sturm et al., 2009; Zhu et al., 2013). FXR could not only inhibit BAs transportation but could also inhibit the formation of liver fibrosis. The specific mechanism is that FXR can inhibit the synthesis of BAs and then further induce BAs detoxification and excretion in small bile duct, increasing bile flow and bile phospholipid content of Bas (Baptissart et al., 2013; Gardes et al., 2013; Matsubara et al., 2013).

Therefore, the nuclear receptor FXR is currently considered to be the hub of cholestasis therapy (Kim et al., 2009). The heterodimer combined with target genes needs to be translated into proteins and then plays biological functions. More importantly, during the process of BA transport, BSEP mainly regulated by FXR plays an important role. BSEP can pump univalent BAs into the bile duct and ultimately into the gallbladder. As the BA receptor, FXR can regulate the gene expression involved in BA metabolism and up-regulate BSEP expression to maintain BA homeostasis in the case of BA overload (Mazuy et al., 2015). It is worth noting that the BSEP promoter contained a response element of FXR (Ananthanarayanan et al., 2001), and the expression of BSEP was significantly decreased in FXR knockout mice (Sinal et al., 2000), which is suggesting that BSEP is a downstream target gene of FXR.

Thus, the selection of FXR downstream molecules is closely related to BA transportation through excited or inhibited FXR. BAs are difficult to separate from the blood and Balasubramaniyan et al. (2013) demonstrated that BSEP was the target gene of FXR and when FXR was up-regulated or down-regulated; BSEP was correspondingly increased or decreased with no natural agonists, i.e., BAs. So the researchers made efforts to intervene hepatic FXR with chemicals or genetic tools to identify the role of FXR on BSEP. The use of FXR agonists, such as GW4064 and 6-ECDCA, can significantly promote liver cells’ uptake of bile salts to reduce cholestasis and then restore bile flow and reduce serum cholestatic indicators (Ye et al., 2009; Zollner and Trauner, 2009). Some reports showed that Fxr-knockout mice had excessive levels of BAs, cholesterol and triglycerides (Anakk et al., 2011). The FXR antagonist guggulsterones has been proven to exacerbate cholestasis in liver cells (Zhao et al., 2014). Moreover, the FXR agonist GW4064 can reduce estradiol-induced cholestasis in liver cells (Seok et al., 2014) to increase uptake of bile salts, restore the flow of bile and reduce the levels of serum bile salt. These compounds have been approved for the treatment of PBC and NASH (Neuschwander-Tetri et al., 2015). Therefore, in order to observe whether emodin could continue to promote the FXR signal pathway in the condition of low and high FXR levels, we chose guggulsterones/siRNA and GW4064/lentivirus to down- or up-regulate FXR. There is another natural product, Dioscin is also can rescue intrahepatic cholestasis in ANIT-Induced rat model through regulating transporters, apoptosis, and oxidative stress (Yao et al., 2017) and through regulating Oatps, Mrp2, and Bsep expression (Zhang et al., 2016).

Therefore, in our experiment, we choose BSEP as our target gene for FXR and choose the FXR inhibitor guggulsterone (Zhao et al., 2014) and siRNA to down-regulate and the FXR agonist GW4064 and lentivirus transfection to up-regulate FXR in our cell experiment. For the LO2 cell experiment, the mRNA and protein expressions of FXR and BSEP were significantly elevated in the emodin group compared with control group. Then, the LO2 cells were interfered with guggulsterones or FXR-siRNA to inhibit the FXR. When compared with the guggulsterones/FXR-siRNA group, the mRNA and protein expressions of FXR and BSEP were also significantly elevated in the emodin groups. Furthermore, to confirm the efficacy of emodin after FXR was up-regulated, we interfered LO2 cells with GW4064 or lentivirus to activate the FXR. When compared with the GW4064/lentivirus-up group, the mRNA and protein expressions of FXR and BSEP were also significantly elevated in the emodin groups. GW4064 can significantly reduce the content of triglyceride, cholesterol and free fatty acid induced by high-fat diet in liver (Ma et al., 2013). Therefore, we speculate that GW4064 could weaken the effect of emodin in promoting the FXR pathway through the degradation of organic solvent that dissolved emodin in this experiment. *In vitro* experiments, we use the normal, guggulsterone stimulation, GW4064 stimulation and FXR-siRNA or lentivirus-up methods to compare the emodin’s positive effect on FXR and BSEP expression, and the results proved that emodin could increase the level of FXR and BSEP, no matter of the basic level of FXR low or high. So it seems like that emodin is an indirect mediator to regulate the expression of FXR. And we verity our idea in rat primary hepatocytes experiment, emodin could also increase the level of FXR and BSEP in rat primary hepatocytes. The FXR-BSEP pathway was activated in ANIT-induced cholestasis rats. After model establishment and treatment, the liver pathological changes and the serum TBIL, DBIL, TBA, ALP, GGT, ALT, and AST were monitored. By emodin intervention, it could be observed that improvement of the hepatic impairment was achieved. The molecules of FXR and BSEP decreased significantly in the model group. After treatment of emodin, the FXR pathways in cholestatic hepatitis were markedly activated, and FXR and BSEP were promoted to some degree.

## Conclusion

This study demonstrated that BSEP is a viable therapeutic target for BA canalicular export and certified the efficacy of emodin in activating the BSEP signaling pathway to alleviate cholestasis *in vitro* and *in vivo*, which may help find a new way to prevent and treat intrahepatic cholestasis. BSEP, the key target molecules that we study may provide new ideas for future precision medicine accurate treatment of intrahepatic cholestasis. Further research is required to demonstrate whether emodin could specifically affect the BA synthesis and detoxification through NTCP (Na^+^/taurocholate co-transporting polypeptide), ASBT (apical Na^+^-dependent BA transporter), OST α-β (organic solute transporter α-β), OATPs (organic anion transporting polypeptides), MDR3 (multidrug resistance protein 3), SHP or UGT2B4.

## Ethics Statement

The study was reviewed and approved by the Research Ethics Committee of Tongji Medical College, Huazhong University of Science and Technology.

## Author Contributions

LZ takes responsibility for the integrity of the work as a whole, from inception to published the manuscript. X-LX and YD conceived and designed the experiments. Z-LC, YW, and PL performed the experiments. HQ analyzed the data. L-SZ, L-LZ, and JH contributed reagents, materials, and analysis tools. YW, Z-LC, and LZ wrote the manuscript. LZ edited the manuscript. All authors approved the final version of the manuscript.

## Conflict of Interest Statement

The authors declare that the research was conducted in the absence of any commercial or financial relationships that could be construed as a potential conflict of interest.
